# A Personalized Voice-Based Diet Assistant for Caregivers of Alzheimer Disease and Related Dementias: System Development and Validation

**DOI:** 10.2196/19897

**Published:** 2020-09-21

**Authors:** Juan Li, Bikesh Maharjan, Bo Xie, Cui Tao

**Affiliations:** 1 North Dakota State University Fargo, ND United States; 2 University of Texas Austin, TX United States; 3 University of Texas Health Science Center at Houston Houston, TX United States

**Keywords:** Alzheimer disease, dementia, diet, knowledge, ontology, voice assistant

## Abstract

**Background:**

The world’s aging population is increasing, with an expected increase in the prevalence of Alzheimer disease and related dementias (ADRD). Proper nutrition and good eating behavior show promise for preventing and slowing the progression of ADRD and consequently improving patients with ADRD’s health status and quality of life. Most ADRD care is provided by informal caregivers, so assisting caregivers to manage patients with ADRD’s diet is important.

**Objective:**

This study aims to design, develop, and test an artificial intelligence–powered voice assistant to help informal caregivers manage the daily diet of patients with ADRD and learn food and nutrition-related knowledge.

**Methods:**

The voice assistant is being implemented in several steps: construction of a comprehensive knowledge base with ontologies that define ADRD diet care and user profiles, and is extended with external knowledge graphs; management of conversation between users and the voice assistant; personalized ADRD diet services provided through a semantics-based knowledge graph search and reasoning engine; and system evaluation in use cases with additional qualitative evaluations.

**Results:**

A prototype voice assistant was evaluated in the lab using various use cases. Preliminary qualitative test results demonstrate reasonable rates of dialogue success and recommendation correctness.

**Conclusions:**

The voice assistant provides a natural, interactive interface for users, and it does not require the user to have a technical background, which may facilitate senior caregivers’ use in their daily care tasks. This study suggests the feasibility of using the intelligent voice assistant to help caregivers manage patients with ADRD’s diet.

## Introduction

### Problem

The progressive brain disorder of Alzheimer disease (AD) causes brain cells to degenerate and die slowly; degrades memory and thinking skills; and, eventually, destroys the ability to perform the simplest tasks of daily life [1]. AD is the most common form of dementia worldwide and the sixth leading cause of death in the United States [2]. In most people with AD, symptoms first appear in their mid-60s [3]. Because the number and proportion of older persons is increasing dramatically [4], estimates suggest that more than 5.5 million Americans, most of them 65 years or older, may have AD [2]. The current growth of the population 65 years and older is one of the most significant demographic trends in US history [2], prompted by the aging of the baby boomers, who in 2030 will be 66-84 years of age and will number 61 million people [5].

People with AD and related dementias (ADRD) are usually cared for by family members or friends, most commonly in their own homes. According to the Centers for Disease Control and Prevention, in 2019, more than 16 million Americans provided about 18.5 billion hours of unpaid care for family and friends with ADRD [6]. Approximately one-third of dementia caregivers are 65 years or older, and approximately one-quarter of dementia caregivers are “sandwich generation” caregivers, people who care not only for an aging parent but also for children younger than 18 years [6]. ADRD caregivers provide care for a longer duration than caregivers of people with other types of conditions [7]. Taking care of a family member with ADRD is overwhelming, and it is important to help caregivers care not only for patients but also for themselves.

### Existing Digital Tools for Healthy Diet

So far, there is no cure for ADRD. Limited medical treatments are available for ADRD’s symptoms, but good care from family caregivers can make the biggest difference in patients’ quality of life [8]. Proper food and nutrition are one of the easiest, most effective, and potentially most enjoyable ways of preventing ADRD and slowing its development [9]. Poor nutrition can increase behavioral symptoms and cause weight loss, and there are many recommendations and guidelines for an ADRD diet [10-13]. However, given the overwhelming amount of available information, it is difficult for the busy caregiver to decide exactly what is best for the patient. In addition, it is not easy for caregivers to remember all the guidelines and constraints. Websites and mobile apps are available for nutrition coaching and meal planning [14,15], but they are not well accepted by ADRD caregivers for several reasons: these tools do not specifically target people with ADRD; their recommendations tend to be generic without considering patients’ specific preferences, comorbidities, culture, or traditions; the tools do not consider caregivers’ time limits for preparing meals or their economic ability to afford food items; and the complexity of such digital tools makes them intimidating for ADRD caregivers, especially older adults who struggle in using computers or smartphones due to inexperience with technology.

### Voice-Enabled Technology

Voice-enabled technology, which is becoming popular worldwide, offers a potential solution. In the United States, the number of people who use voice assistants such as Amazon’s Alexa or Apple’s Siri is rapidly increasing; in 2019, more than 111 million people in the United States used a voice assistant at least monthly, equivalent to 39% of internet users and 34% of the total US population [16]. Amazon’s Alexa is the most popular voice technology, with a 75% share of the market [17]. This voice interface is capable of performing various tasks and controlling various systems. Currently, interaction and communication with Alexa are available in English, German, French, Italian, Spanish, and Japanese [18].

Alexa’s capabilities can be extended with “voice skills,” apps developed by third-party vendors. As of March 23, 2020, there were more than 70,000 skills available. These “skills” enable users to interact with their Alexa-enabled devices using natural language in a variety of ways including gaming; shopping; and gathering information about news, weather, travel, health, and fitness.

As voice-activated technology has become increasingly popular, researchers and engineers have begun to develop it for use in health care domains, ranging from health news briefs, nutritional guides, and fitness trackers to programs for meditation and yoga. The Mayo Clinic, for example, has launched a “First Aid” Alexa voice skill that answers questions about how to treat common mishaps such as cuts and burns [19]. It offers self-care instructions for first aid, as well as guidance on when a person should seek emergency help.

### Objectives

In this paper, we present the design, implementation, and preliminary testing of an artificial intelligence–powered voice assistant to provide personalized education and guidance for caregivers on food, nutrition, and cooking for a loved one with ADRD. This voice assistant gives the ADRD caregiver continuous access to useful tips about food, nutrition, and eating behaviors. It also recommends food and meals. The tips and recommendations are personalized, specific to the patients with ADRD’s condition including the patient’s ADRD stage, preferences, and medical conditions. Moreover, the assistant’s recommendations are specific to the caregiver, based on the caregiver’s time limits, financial ability, and education level. Because voice is our most common mode of communication, a voice assistant provides a natural way to engage with technology that requires minimal training. This is especially valuable for caregivers who are older adults, who account for more than one-third of caregivers, and who may find it difficult to use other forms of technology that require fine motor skills, hand-eye coordination, or good vision.

## Methods

### System Overview

The goal of our project is to design a voice-enabled intelligent assistant to support effective diet management for ADRD caregivers. The voice assistant is built on top of a comprehensive personalized ADRD diet care knowledge base, a semantics-based knowledge graph reasoning engine, and Amazon’s cloud-based voice service Alexa.

**Figure 1:**
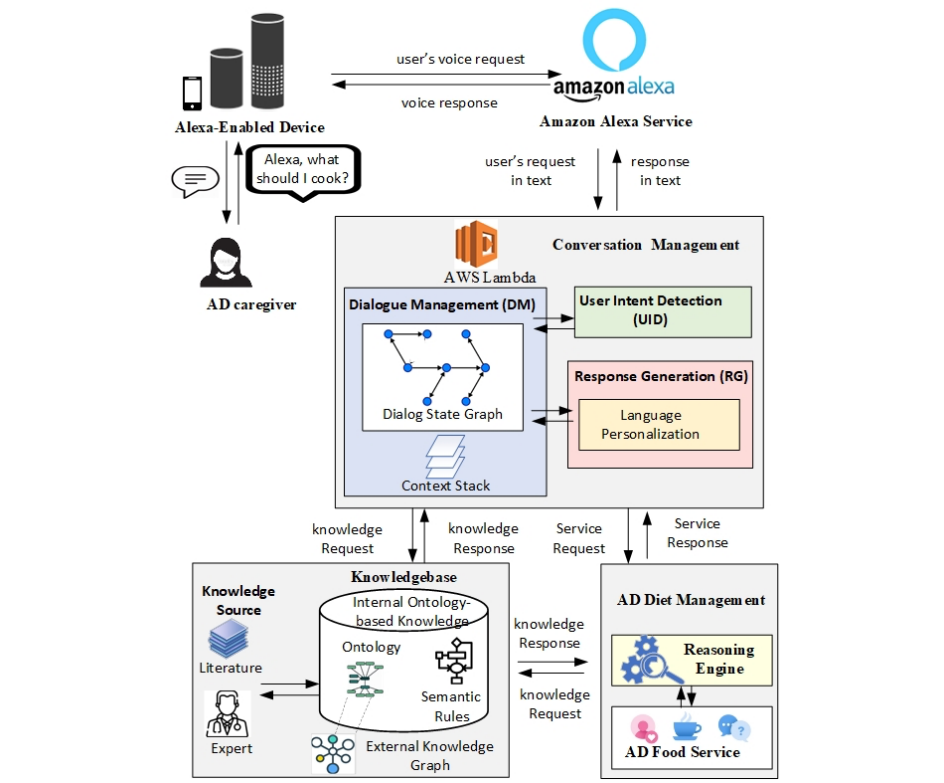
Architecture of the system. AD: Alzheimer disease; AWS: Amazon Web Services.

Figure 1 illustrates the system architecture that enables a caregiver’s access via Alexa. This architecture has three major components: (1) conversation management, which manages conversations with users; (2) a knowledge base, which provides knowledge for the system; and (3) ADRD diet management, which provides detailed personalized diet education, recommendations, and planning services to users. We have designed and implemented these three components as shown in the three boxes in Figure 1. At the same time, we use Alexa’s existing services to recognize the user’s speech, convert the user’s speech to text, and transform the system’s response to speech and respond to the user. The conversation management component includes three major modules: user intent detection (UID), dialogue management (DM), and response generation (RG). The knowledge base component includes internal and external knowledge. The internal knowledge consists of ontologies and rules defined by ontological language. External knowledge includes related existing knowledge that can be reused in the system. ADRD diet management includes a semantic reasoning engine to support customized question answering, education, recommendations, and planning services for food and nutrition.

As illustrated in Figure 1, when the system receives a user’s request, the request is sent to the conversation management component, where it goes through the three major modules of conversation management to produce a response. First, the UID module analyzes the user’s request or command text and matches the text with predefined intent and the dialogue state. Second, the DM module examines the input, executes the dialogue policy, and updates the dialogue state. Based on the user’s intent, the Amazon Web Services’ (AWS’s) Lambda service will call back-end ADRD diet management information corresponding to the intent. This service is supported mainly by a semantics-based reasoner, working on the user’s input, facts, and rules defined in the knowledge base. The semantic reasoning links the dialogue with the user’s (ie, the caregiver’s and patient’s) profile, context, and ADRD medical guidelines [10-12,20-23], enabling personalized services. Third, the RG module uses the speech act and content selected by the DM module to build a response. The response language is chosen based on the user’s background such as education.

### Conversation Management

This component allows the user to interact with the voice assistant. It receives the user’s voice input from Amazon Alexa. Alexa converts the user’s speech into text using automatic speech recognition and natural language understanding. The UID module matches the user’s voice, converted to text, with a predefined intent. The intent corresponds to a system action that fulfills the user’s spoken request. To effectively identify the user’s intent, we extend users’ utterances with synonyms and ontology concepts (ie, classes and instances) from our knowledge base. Because the system focuses on diet management, we have chosen top-level food-, nutrition-, and diet-related classes, and classes or instances that are connected to the chosen top-level classes by two major relationships of an ontology: the hierarchical specialization/generalization (or *IS-A*) relationship and the *type* relationship. This allows the DM module to better identify ADRD food management–relevant entities.

**Figure 2:**
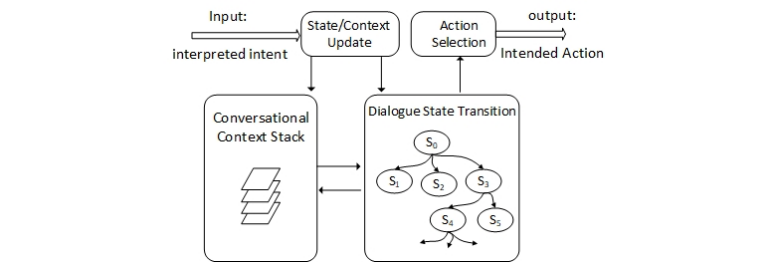
Information flow of dialogue management module.

Figure 2 illustrates the information flow of the DM module. The interpreted user’s intent is the input to the module. The intent corresponds to an action that fulfills the user’s spoken request. Based on our requirement analysis, in our prototype system, we have predefined five categories of intents such as providing tips on proper diet and suggestions for healthy meals. When a user speaks to the voice assistant (asks a question, requests a service, or answers a question), the voice assistant elicits required information or clarifies unclear information. To determine what questions the virtual assistant should ask, in what order, and when, we use a finite-state approach to model the dialogue structure. A state transition graph is designed for every type of service request. This graph can model and track context, different dialogue states, and corresponding transitions to maintain the flow of the dialogue. It includes an initial state, S0, representing the start of the conversation, and a set of successor states for any particular state (eg, S4 and S5 are successors of S3) that may result from input messages. Incoming messages from the user will set the conversation to a new state and a corresponding response. The next state can be calculated based on the dialogue history, the input from the user, and the conversation’s context. Dialogue history is recorded as the path of states from the initial state to the current state. The graph helps the system produce coherent responses within an ongoing conversation. It also guides the conversation’s direction toward a predefined schema instead of letting the conversation’s topic drift randomly or letting the conversation be controlled by the user.

In a dialogue session, context information is integrated with dialogue states to determine the next potential state. A short-term context will be kept for a few dialogue states, but a long-term context may be kept permanently. For example, a patient with ADRD’s age, ADRD stage, food preferences, and allergies constitute long-term context information; the meal a caregiver chooses for lunch can be short-term context information. Different context information may have different expiry times. The topic of the last conversation and the last conversation state in the dialogue state graph are also stored in the session context.

After an action is determined on the basis of the dialogue state graph, it is returned to the RG module. At each turn of conversation between the user and the voice assistant, the DM module performs a series of steps to collect all the required data (slots) from the user. It then tries to find the response that both addresses the user’s request and meets the constraints of the conversation topic and context. The response requires knowledge support from the back-end AD diet management component. This component bridges the gap between high-level application logic and low-level dialogue specifications. It allows the dialogue to be dynamically adapted according to the current high-level context.

### Knowledge Base

The voice assistant’s “brain” is a comprehensive knowledge base, which stores the knowledge that informs the answers, recommendations, and tips that the voice assistant provides. To construct this knowledge base, we conducted an extensive review of the scientific literature on ADRD home care and on food and nutrition tips for ADRD care [10-12,20-23]. We used an ontology-based information model to fuse heterogeneous-related information and knowledge in constructing the knowledge base, thus extending Alexa’s knowledge base by adding new customized knowledge. This ontology focuses on concepts, relations, constraints, and rules about general care for patients with ADRD, diet guidelines for ADRD, each patient’s profile, and each caregiver’s profile. We thus enrich and extend our knowledge with existing (semi)structured knowledge, for example, by adding knowledge related to disease (Unified Medical Language System [24], SNOMED Clinical Terms [25], International Statistical Classification of Diseases and Related Health Problems, 10th revision [26]), drugs (DrugBank [27]), and food and nutrition (United States Department of Agriculture’s [USDA’s] FoodData Central [28]). Currently, we have integrated only the USDA’s FoodData Central for detailed food and nutrition knowledge within the system. This food and nutrition information, together with the ADRD diet guidelines that we have collected [10-13,20-23], should be able to address most of the proposed services required by the voice assistant. This is an ongoing project; we are working to enrich the voice assistant’s functionality to support other aspects of ADRD care, which may benefit from other knowledge sources.

**Figure 3:**
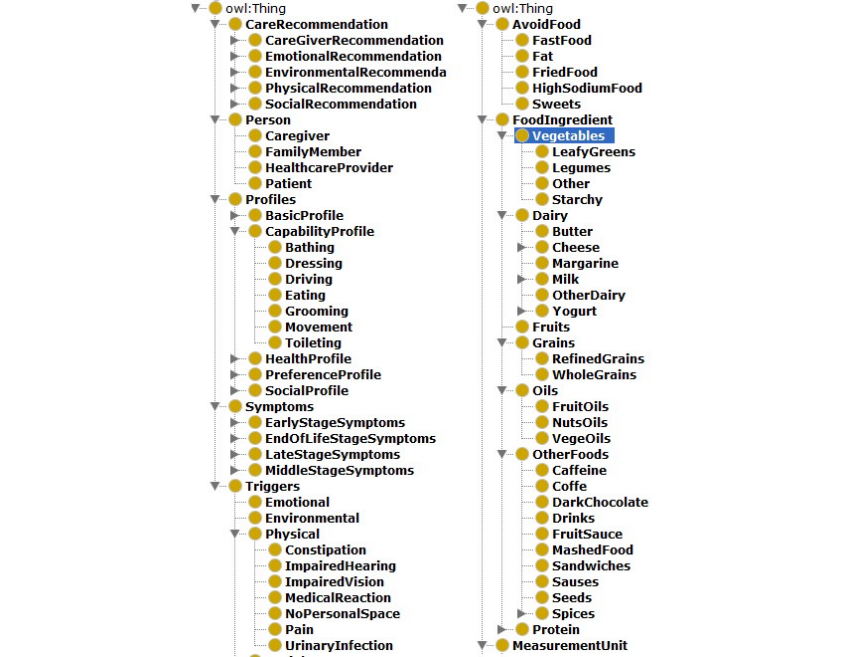
Part of the classes defined in the ontology used in the knowledge base.

An ontology represents a domain of discourse explicitly. It defines the concepts and relationships used to describe and represent the domain [29]. It defines domain knowledge semantically in standard ways that allow comprehensive, transportable machine understanding. The knowledge base mainly uses three ontologies: (1) a user profile that captures both the patient’s and the caregiver’s social, economic, cultural, and physical properties, and provides evidence for personalization; (2) ADRD diet support and education, which captures essential knowledge in a user-friendly language for healthy eating; and (3) food and nutrition knowledge extracted from other sources [30] but reorganized for our application’s purpose. Figure 3 shows part of the system’s high-level ontology.

Besides concepts (classes) and relationships (properties) defined in the ontology, the knowledge base includes rules based on professional medical guidelines related to AD diet. We have collected AD general caring guidelines and food and nutrition guidelines such as from the National Institute on Aging [23], Alzheimer’s Association [22], Mayo Clinic [31], alzheimers.net, the National Heart Foundation of Australia [20], and the USDA [32]. The collected rules were further verified by two experienced clinicians on our research team who specialize in the diagnosis and management of cognitive disorders including ADRD and were then converted to rules that computers can “understand.” We use the Semantic Web Rule Language (SWRL) [33], an expressive World Wide Web Consortium standard Web Ontology Language (OWL)–based rule language to present the generated rules.

### AD Diet Management

The back-end ADRD diet management component receives the user’s request as input and calls the corresponding back-end service to generate a response that is sent back to the RG module in the conversation management component. The back-end services are built to enable context-aware personalized recommendations, education, and question answering. The foundation of this component is a description logic (DL) [34] query-answering and reasoning engine over the OWL ontology and its extended knowledge graph. In particular, we use DL to check the satisfiability of the whole knowledge base and answer complex queries (eg, unions of conjunctive queries) over the knowledge base. We separate the ontology into TBox (classes and properties) and ABox (instances) [35]. Therefore, reasoning can be performed on two different levels, the TBox level and ABox level. This improves reasoning performance by avoiding complex operations over a large number of ABox instances. Instead, most of the queries can be performed using the Structured Query Language engine over the ABox triple store.

A backward-chaining algorithm is used for automatic reasoning. Backward chaining [36] is a goal-driven reasoning process. To prove a goal, all of its subgoals need to be recursively proved. The reasoning engine uses data in the knowledge base to match the initial goal by matching rules for true consequent and then assuming true antecedent. As an example, two class subsumption rules are given in equations 1 and 2.

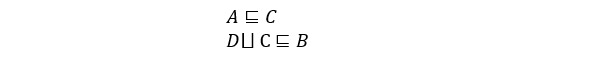



Supposing a being of class D and A, the following backward chaining application in equation 3 illustrates how to prove a being of class B.

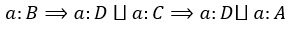



The reasoning engine takes the AD diet management rules in SWRL format [33], facts from food and nutrition ontology, and the patient with ADRD’s and caregiver’s profile information from the profile ontology in the knowledge base. When a user’s query or request is passed to the back end from the DM module, the contextual information and auxiliary information such as intent and sentiment are fed into the query and reasoning engine together with the rules and facts to control the generation of responses. The goals (and subgoals) always match the affirmed versions of the consequents of implications, and their antecedents are then considered as new goals, which ultimately must match known facts (eg, in the previously mentioned example, a being of class D and A). The backward chaining–based reasoner links the dialogue between the user and voice assistant with the profile of the particular caregiver and patient, other context, and ADRD diet medical guidelines. In this way, the system can provide context-aware personalized recommendations.

## Results

### Prototype Implementation

We have implemented a prototype of the voice assistant as an Amazon skill “ADRD-DietAssistant.” We have conducted a set of preliminary evaluation tests to assess the voice assistant’s technical viability and effectiveness.

- Front-end implementation:
  Alexa Skills Kit (ASK):
    Dialogue design, intent definition, utterances to activate intents, slot customization
  The Amazon Lambda service:
    Runtime: Node.js 12.14.1
    Software development kit (SDK) to communicate with Alexa: ASK-SDK
    Logging service: Amazon CloudWatch Logs
- The back-end diet management server is configured as follows:
  Processor: Intel Core i-7-4770 CPU @ 3.40 GHz (8 CPUs), ~ 3.4 GHz
  RAM: 32 GB
  Operating system: Windows 10 Home 64-bit (10.0, Build 17763)
  Programming Language: Java 8, Java Development Kit 1.8
  Semantic service: OWL Application Programming Interface and SparkJava
  Database: MySQL: The back-end AD Diet Management

### Evaluation Outcomes

#### Use Cases

The voice assistant can support a variety of food and nutrition recommendations, education, and planning services including tips on proper diet, handling challenges in eating, food item and nutrition explanations, meal suggestions, and recall of daily and weekly diet histories. In a conversation, different service categories can be integrated to serve the user’s requests.

**Figure 4:**
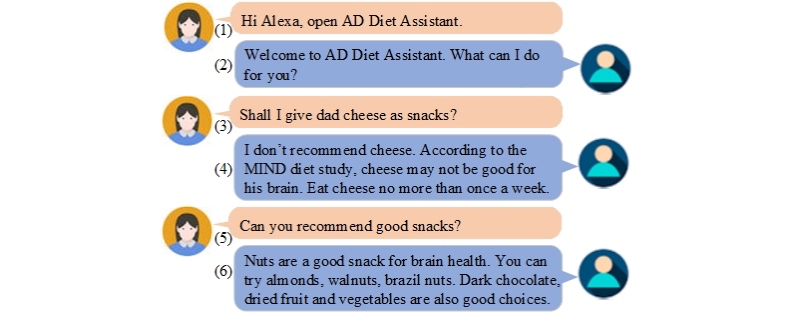
An example of a conversation between the user and the voice assistant about food and nutrition education, and meal suggestion. AD: Alzheimer disease; MIND: Mediterranean–Dietary Approaches to Stop Hypertension Intervention for Neurodegenerative Delay.

Figure 4 demonstrates one example of a conversation. The user is a female caregiver; she is taking care of her 65-year-old father, who is in early-stage ADRD with type 2 diabetes. In this conversation, the user asks the voice assistant about the suitability of food items and gets the right food recommendation.

Utterance 1 is an invocation that activates the diet assistant voice skill. Once the skill is activated, the voice assistant greets the user (Utterance 2). A conversation then follows based on the user’s requests. The voice assistant triggers a specific intent designed to handle the user’s specific request. In Utterance 3, the user asks the voice assistant whether a certain food is good for the patient. This triggers the back-end service to check whether the food item—cheese in this case—is good as a snack or not. The variables collected from this utterance are “cheese” and “snack,” which are passed on to the system’s back end. The system’s back end has an intent handler that then checks to determine whether “cheese” is good as a “snack” for people with Alzheimer and diabetes, using facts and rules stored in the ontological knowledge base. It then prepares a speech to be sent back to the user as a response, as shown in Utterance 4. In Utterance 5, the user asks for a good snack suggestion. The back end then uses intent chaining to find a healthy “snack.” The response is shown in Utterance 6. 

**Figure 5:**
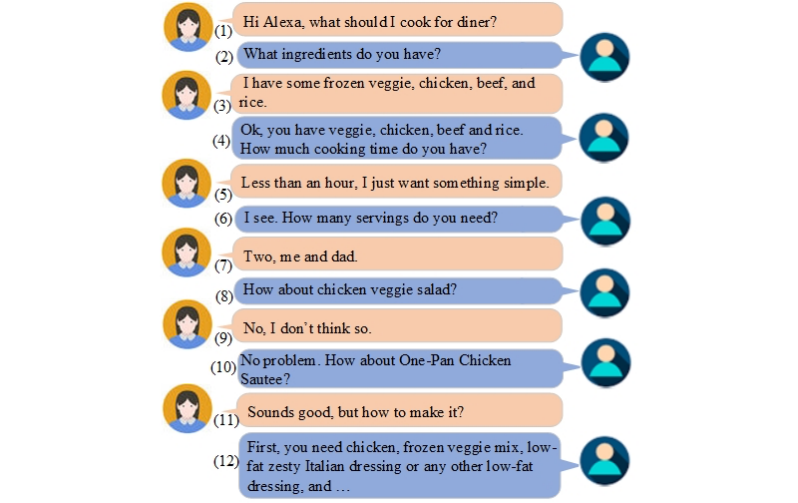
An example fragment of a conversation between the user and the voice assistant about meal recipe suggestion.

Another sample conversation is shown in Figure 5. In this conversation, the voice assistant recommends a healthy recipe to the caregiver in response to her request, the context of the conversation, the patient’s profile, and healthy eating guidelines. Utterances 2-7 show how the voice assistant acquires the information to make an appropriate recommendation so that it can prepare all the slots to call the back-end intent. Utterance 7 shows that a user may provide more information than was requested by the voice assistant. The voice assistant gives a recommendation in Utterance 8. The user rejects the suggestion in Utterance 9. Utterance 10 shows that the voice assistant acknowledges the rejection and provides an alternative suggestion. In Utterance 12, the voice assistant presents the recommendation.

To provide a satisfying response to a user’s requests, the back-end diet management server will consider multiple factors that affect the patient’s diet decisions, such as ADRD nutrition requirements, food availability, cooking time, servings, preferences, medical restrictions, and cultural and religious constraints. The system extracts all these constraints from the user’s profile, context, and health guidelines.

For the recipe recommended in Figure 5, the system follows multiple guidelines in the knowledge base, including the following:

- The Mediterranean–Dietary Approaches to Stop Hypertension Intervention for Neurodegenerative Delay (MIND) diet [10] encourages the consumption of all kinds of vegetables, berries, nuts, olive oil, whole grains, fish, beans, poultry, and a moderate amount of wine.
- The MIND diet also encourages limiting one’s consumption of butter and margarine, cheese, red meat, fried food, pastries, and sweets.
- The US Department of Health and Human Services Dietary Guidelines [32] state that if a person consumes three meals per day, one meal should contain 800-850 calories for a man.
- The American Diabetes Association Diabetes Guidelines [37] state that a person’s dinner total calories should be 25% of the person’s Estimated Energy Requirement, protein intake should be 20%-30% of meal energy, and sugar should be less than 10% of meal energy.

The voice assistant also checks for other factors affecting the user’s diet decisions, such as the user’s (patient’s or caregiver’s) preferences, allergies, religious constraints, and so forth.

#### Qualitative Evaluation

We have performed a set of qualitative evaluations to test the system’s performance. The testers, who included one faculty member and four graduate students on our research team, performed 180 conversations with the voice assistant in our research lab. These conversations were invoked by the testers’ requests. Each of the requests belonged to one of the following service categories: food item and nutrition explanations, meal and recipe suggestions, and tips for proper diet. Otherwise, testers had maximum freedom and flexibility in talking to the voice assistant.

In our testing, we defined a threshold, the maximum number of turns in the dialogue between the user and the voice assistant, to limit the dialogue’s length. If a conversation exceeded this limit and the user did not receive a satisfying response, the conversation was noted as failed. In our evaluation, we set the threshold of maximal dialogue turns at 20. Given this threshold, the dialogue success rate is defined by equation 4:

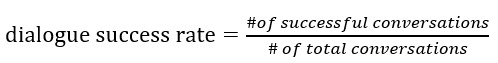



The correctness of information provided by the voice assistant is defined in equation 5. Correctness is determined by manually checking whether the recommended content is correct on the basis of the medical guidelines and consistent with a user profile artificially generated for the test, that is, whether the recommendation satisfies the user’s request and is consistent with the patient’s and caregiver’s profile, following the constraints and guidelines in the knowledge base:

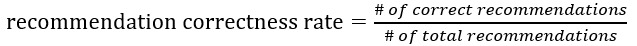



Table 1 shows the test results. The dialogue success rate ranged from 80% to 100%. Failed dialogues occurred when the voice assistant could not match the user’s intention with back-end services—when it could not relate the user’s utterance with predefined intents (function) and the slots (parameters) required to call the intents. For recipe recommendations, most failed conversations occurred when the voice assistant could not provide a satisfying recipe at the beginning. Failure also occurred when matching knowledge was not found in the knowledge base. Although Alexa can effectively match what is said by the user against all possible combinations of defined utterances and slot samples, it has a limited capability in matching an undefined utterance to a slot.

**Table 1:** Dialogue performance test.

Metrics/purpose	Category 1	Category 2	Category 3
Average dialogue time (sec)	29.7	68.8	45.7
Time per turn (sec)	6.75	6.68	6.34
Number of turns per dialogue	4.4	10.3	7.2
Dialogue success rate, n/N	51/59	48/60	61/61
Recommendation correctness rate, n/N	51/51	48/48	61/61

## Discussion

### Principal Results

Healthy eating may help slow and even prevent the progression of ADRD and subsequently improve the health status and quality of life in older age [21]. To assist healthy eating, we have designed, developed, and evaluated a personalized, actionable, and engaging voice assistant to help ADRD caregivers manage patients’ diet. Building on an ontology-based knowledge base extended with external knowledge graphs and a reasoning and query-answering engine, the voice assistant can answer user’s questions and provide personalized and refined food and nutrition recommendations. It follows evidence-based clinical guidelines and takes into account health constraints and personal preferences of both caregivers and patients. The voice assistant has been evaluated using various types of cases. Preliminary test results have demonstrated reasonable rates of dialogue success and recommendation correctness. This study provides preliminary evidence that it is feasible and effective to implement a voice-based virtual assistant to provide diet-related services to ADRD caregivers.

### Limitations and Future Work

This study has limitations. The qualitative evaluation was performed within a laboratory setting. Whether or not actual caregivers in a natural setting will use the assistant in ways similar to those of researchers in the laboratory remains for further research. We plan to deploy the voice assistant in a natural setting with ADRD caregivers and patients with early stage ADRD as users. More comprehensive user studies will allow us to evaluate the voice assistant’s usability, users’ satisfaction, and outcomes for improved health and quality of life. We will also conduct focus groups to collect users’ feedback about their knowledge needs to improve our knowledge base.

To increase the dialogue success rate, we propose several approaches to improve the flexibility and accuracy of matching:

- The voice assistant can provide more oral guidance during conversations so that users have a better understanding of the voice assistant’s expectations.
- Developers should define more combinations of utterances and synonyms for each intent and its slots to improve the matching rate.
- Although Amazon uses machine learning to match a user’s intention with defined intent, this learning is limited, and matching performance largely depends on keywords and sentences provided at the design stage. We will design our machine learning algorithms based on domain knowledge and user feedback to improve accuracy of understanding.
- We will extend our knowledge base with more relevant knowledge.

In addition, we plan to capture conversations and use them for machine learning to improve conversation accuracy. If we can solve potential issues of privacy and security with users’ approval, in the future, we will collect and use large amounts of conversation information for increased accuracy.

### Conclusions

Voice is the most natural and powerful mode of human communication. Our voice assistant gives users a natural, interactive interface, and it does not require the technical background that might be a barrier to senior caregivers’ use of new technologies for daily care tasks. This may enhance caregivers’ engagement in ADRD diet care and reduce caregiver burden, improving the care of patients as well as caregivers’ own health and well-being.

## Abbreviations

AD: Alzheimer disease
ADRD: Alzheimer disease and related dementias
ASK: Alexa Skills Kit
DL: description logic
DM: dialogue management
MIND: Mediterranean–Dietary Approaches to Stop Hypertension Intervention for Neurodegenerative Delay
OWL: Web Ontology Language
RG: response generation
SDK: software development kit
SWRL: Semantic Web Rule Language
UID: user intent detection
USDA: United States Department of Agriculture
